# A Fuzzy Control Strategy for Multi-Goal Autonomous Robot Navigation

**DOI:** 10.3390/s25020446

**Published:** 2025-01-14

**Authors:** Stavros Stavrinidis, Paraskevi Zacharia, Elias Xidias

**Affiliations:** 1 Department of Industrial Design and Production Engineering, University of West Attica, Egaleo, 12241 Athens, Greece; s.stavrinidis@uniwa.gr; 2 Department of Product & Systems Design Engineering, University of the Aegean, 84100 Syros, Greece; xidias@aegean.gr

**Keywords:** mobile robot, navigation, sensors, path planning, collision avoidance, genetic algorithms, fuzzy controller, TSP, CoppeliaSim

## Abstract

This paper addresses the complex problem of multi-goal robot navigation, framed as an NP-hard traveling salesman problem (TSP), in environments with both static and dynamic obstacles. The proposed approach integrates a novel path planning algorithm based on the Bump-Surface concept to optimize the shortest collision-free path among static obstacles, while a Genetic Algorithm (GA) is employed to determine the optimal sequence of goal points. To manage static or dynamic obstacles, two fuzzy controllers are developed: one for real-time path tracking and another for dynamic obstacle avoidance. This dual-controller system enables the robot to adaptively adjust its trajectory while ensuring collision-free navigation in unpredictable environments. The integration of fuzzy logic with TSP-based path planning and real-time dynamic obstacle handling represents a significant advancement in autonomous robot navigation. Simulations conducted in CoppeliaSim validate the effectiveness of the proposed method, demonstrating robust navigation and obstacle avoidance in realistic environments. This work provides a comprehensive framework for solving multi-goal navigation tasks by incorporating TSP optimization with dynamic, real-time path adjustments.

## 1. Introduction

Mobile robots are autonomous systems designed to operate in a wide range of environments without direct human intervention. Equipped with various sensors, actuators, and computational components, these robots can perceive their surroundings, make decisions based on sensory input, and execute actions to accomplish specific tasks. Their applications span numerous fields, including industrial automation, logistics, the exploration of hazardous areas, and personal assistance. The increasing versatility of mobile robots is a direct result of advances in robotics, artificial intelligence, and computer vision, enabling them to operate more efficiently and adapt to different scenarios.

One of the most complex challenges in mobile robotics is multi-goal navigation, particularly in environments with static and dynamic obstacles. Efficient and safe navigation requires advanced techniques such as localization, mapping, and path planning, which enable the robot to determine its position, model its environment, and compute optimal, collision-free routes. The problem of determining the shortest collision-free path that connects a set of specified goal points within the robot’s operational environment is known as the multi-goal path planning (MGPP) problem [1,2,3], a complex variation of the NP-hard traveling salesman problem (TSP). MGPP involves two key tasks: first, optimizing the sequence in which the goal points, situated in the free workspace, should be visited; and second, calculating the shortest collision-free path between these points.

In the context of MGPP, determining the optimal sequence of goal points within the robot’s workspace is a variation of the traveling salesman problem (TSP), with the added complexity of requiring navigable and collision-free paths. This requirement for safe traversal between goal points significantly increases the complexity of MGPP compared to the standard TSP, as it must account for the presence of static and dynamic obstacles. Recently, TSP-based approaches have gained significance in robotic multi-goal navigation due to their efficiency in optimizing travel routes while minimizing travel distances and avoiding obstacles. To address these challenges, both online and offline path planning methods have been developed—online techniques rely on real-time sensor data to adapt to dynamic environments [4,5], while offline approaches use pre-existing environmental information [6].

The multi-goal path planning (MGPP) problem has been extensively studied, with many proposed approaches driven by practical applications such as planning for robotic arms [2], industrial manipulators [7], coordinate measuring machines (CMMs) [8,9], rescue missions [10], and pick-up and delivery tasks [11]. In the realm of mobile robotics, Genetic Algorithms (GAs) have been frequently applied to solve the TSP due to their robust optimization capabilities [1,12,13,14]. The GA, an adaptive search technique inspired by natural selection and genetic reproduction, is widely employed to conduct random searches for the solving of complex optimization problems.

This paper addresses the challenge of determining the optimal tour for multi-goal navigation while ensuring collision-free movement in the presence of both static and dynamic obstacles. It presents a fuzzy logic-based control strategy designed for autonomous navigation in environments where obstacles include unpredictable and dynamic objects. The proposed approach involves the development of an intelligent control system utilizing fuzzy logic. The key contributions of this work can be outlined in four main aspects:

1. A novel control strategy is developed to address the multi-goal navigation problem for mobile robots in environments with both static and dynamic obstacles. This problem is classified as NP-hard due to its connection to the TSP;
2. An advanced path planning algorithm is implemented to enhance the process of determining optimal trajectories, strongly enhancing the efficiency and effectiveness of autonomous robot navigation;
3. Two fuzzy controllers capable of handling the dynamic aspects of the navigation problem are designed. These controllers operate in real-time applications, ensuring responsive and adaptive behavior;
4. A robust path planning algorithm for static obstacle avoidance combined with fuzzy controllers for dynamic obstacle navigation is integrated to ensure smooth and collision-free navigation, significantly enhancing the robot’s ability to navigate complex and unpredictable environments;
5. The solution addresses a global optimization problem by combining two NP-hard problems: the TSP within the robotic context and real-time collision-free path planning. This integrated approach offers a robust framework for tackling both static and dynamic navigation challenges.

The structure of this paper is as follows: Section 2 provides a review of the related literature. Section 3 presents robot kinematics and outlines the problem being addressed. Section 4 details the path planning framework designed for collision avoidance. Section 5 introduces the key concepts related to autonomous multi-goal navigation and analyzes the developed fuzzy controllers for navigation toward the goal and for collision avoidance in a complex environment. Section 6 presents the experimental results obtained through simulations in the CoppeliaSim (Edu version 4.5.1) environment. Lastly, conclusions and suggestions for future research directions are made in Section 6.

## 2. Literature Review

In the following paragraphs, key studies on recent advancements and methodologies are presented that address challenges in obstacle avoidance, the optimization of navigation paths, and adaptive control strategies.

The work in [15] presents a novel approach for robotic path planning in dynamic and pedestrian-heavy environments like airports. The authors formulate the problem as a variation of the traveling salesman problem (TSP), incorporating a potential field to account for obstacles and pedestrians. They introduce a socially compliant path planning scheme using a Self-Organizing Map (SOM) to optimize the robot’s route while minimizing disturbances to nearby pedestrians. Their approach successfully combines social awareness with efficient path planning, demonstrating its effectiveness in simulation environments.

The authors in [16] investigate the use of the TSP for optimizing multi-goal navigation tasks. The authors focus on planning paths in uncertain environments using belief space, where the robot’s knowledge of its environment evolves as it gathers data. The study utilizes the TSP to determine the optimal sequence of goal points, addressing challenges posed by incomplete or imprecise information. By incorporating belief space into the planning process, the approach aims to improve the efficiency and adaptability of mobile robots in dynamic environments.

The authors in [17] investigate the MGPP problem, which focuses on finding the shortest collision-free path for robots visiting multiple goal points. The study proposes a two-step solution: first, optimizing the sequence of goal points using a Genetic Algorithm (GA), and second, generating an initial path using the Boundary Node Method (BNM), followed by the Path Enhancement Method (PEM) to minimize the overall path length. The authors also extend their approach to multi-robot systems, ensuring collision avoidance between robots and obstacles. The paper demonstrates its effectiveness through simulations and real-world experiments using a mobile robot.

The work in [18] introduces an approach for indoor mobile robot navigation that integrates an improved A* algorithm, the TSP, and the Dynamic Window Approach (DWA). The authors propose using the improved A* to determine the optimal paths between goal points while removing redundant nodes for a more efficient solution. To optimize the order in which the robot visits multiple goals, the TSP is solved using the Ant Colony Optimization (ACO) algorithm. Additionally, the DWA is applied for real-time obstacle avoidance and adaptive speed control, ensuring smooth and safe traversal for all targets.

The authors in [19] study the problem of autonomous navigation for unmanned surface vehicles (USVs) in complex and unknown environments. The authors enhance the traditional D* Lite algorithm to achieve multi-goal path planning and dynamic obstacle avoidance. Their improvements include expanding the search range to accommodate the limited maneuverability of USVs and using a minimum binary heap to optimize the priority queue, significantly reducing path planning time. The experimental results show the enhanced algorithm’s efficiency in dynamic environments, highlighting its practical application in real-world scenarios. While the study effectively improves global path planning for USVs, it focuses primarily on static environmental modeling and dynamic obstacle avoidance without employing adaptive real-time control strategies.

In [20], the authors present a novel approach to enhance multi-goal motion planning for robots operating in complex, obstacle-rich environments. The authors combine machine learning with TSP solvers and sampling-based motion planning to address the challenges of determining feasible paths and goal sequences. The machine learning models predict distances and directions between locations, incorporating obstacle information and robot dynamics, which are then used by the TSP solver to compute optimal tours. The proposed method was tested on vehicle- and snake-like robot models, demonstrating efficiency and scalability in navigating multiple goals under dynamic constraints. This approach integrates predictive modeling for efficient goal sequencing and focuses on improving path planning without explicitly addressing adaptive real-time collision avoidance with dynamic obstacles.

The work in [21] addresses the problem of autonomous navigation for mobile robots in multi-goal missions, particularly focusing on scenarios with challenging environments. The authors propose the SMUG planner, which integrates a two-stage planning approach combining the TSP solver and Iterative Dynamic Programming (IDP). The SMUG planner aims to generate an efficient and collision-free global path to visit a set of predefined targets while accounting for safety by employing a hierarchical state validity checking mechanism. Implementation in the quadruped robot ANYmal demonstrates the method’s effectiveness, particularly in rough terrains. This approach primarily focuses on global path planning without extensively addressing real-time adaptation to dynamic obstacles.

In [22], the authors present a combined approach for optimizing navigation in agricultural scenarios. It leverages a global planning algorithm based on the TSP and the Capacitated Vehicle Routing Problem (CVRP) to schedule harvest locations effectively. The local planning uses the Informed Rapidly Exploring Random Tree Star (IRRT*) method to navigate complex terrains, addressing challenges posed by obstacles and low-traction surfaces. The study demonstrates improved path smoothness and efficiency in navigating agricultural environments, particularly in avoiding obstacles and slippage zones, making it suitable for precision farming applications. However, while the proposed strategy effectively integrates global and local planning methods, it primarily focuses on static environments with predictable terrain characteristics.

The already published studies have demonstrated effectiveness in handling static and dynamic obstacles, especially in structured or semi-predictable environments. To address the challenges posed by unpredictable and dynamic obstacles, our research work introduces a fuzzy logic-based control system designed to enhance real-time adaptability in highly dynamic and unpredictable environments. The proposed system effectively integrates dynamic obstacle avoidance with path tracking, enabling robots to navigate complex environments with increased flexibility and robustness.

## 3. Preliminaries and Problem Framework

### 3.1. Robot Kinematics

**Figure 1:**
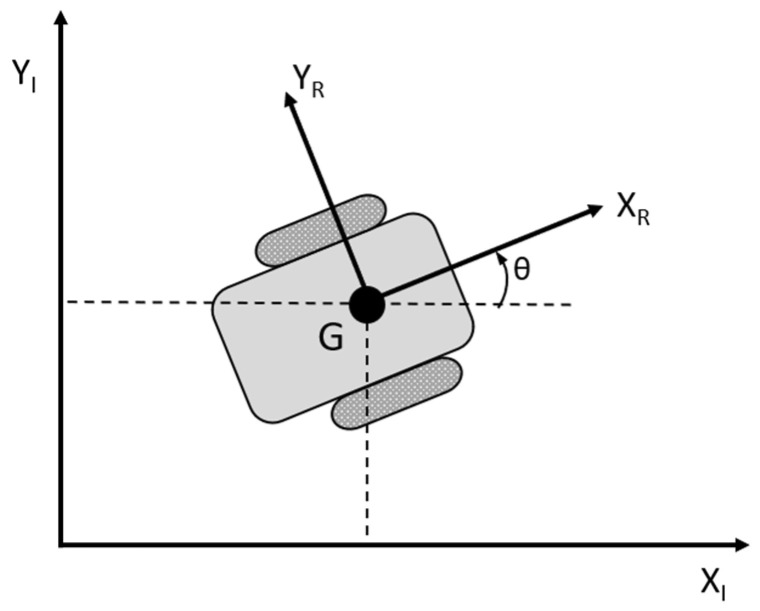
The mobile robot within the global and local reference frames.

The primary objective of this study is to develop a control system that enables the safe navigation of a mobile robot in a dynamic environment while accounting for its kinematic and geometric properties. To describe the robot’s movement, two critical reference frames are established: a global reference frame, fixed in the environment, and a local reference frame, attached to the robot, which is treated as a rigid body (see Figure 1). The robot’s position and orientation are thus expressed as a vector with three elements: $p=\left[\begin{array}{c} x\\ y\\ \theta \end{array}\right]$. This study employs a two-wheeled differential drive robot to create a kinematic model (Figure 1).

The kinematic model of the wheeled robot during motion is based on several key assumptions. First, each wheel remains perpendicular to its plane throughout the movement. Second, there is only one point of contact between each wheel and the surface. Lastly, the wheels are assumed to roll without slipping. In addition to these assumptions, two constraints are applied: the rolling constraint, where the motion component along the wheel’s plane matches the wheel’s rotational velocity, and the slipping constraint, which ensures that the motion component in the direction perpendicular to the wheel’s plane is zero. According to [23], a differential drive system consists of two wheels mounted on the same axis, each powered by separate motors. Since both wheels contribute to the robot’s motion, the overall movement is determined by calculating the contribution of each wheel independently.

### 3.2. Problem Formulation

Assume a mobile robot (in our case, the Pioneer P3-DX model provided by CoppeliaSim) that navigates in a warehouse environment and is requested to visit a set of designated task points to perform tasks (e.g., shelf inspection and safety checks). Shelf inspection concerns checking for damaged or misplaced items and safety checks concern the clearance of emergency exits, hazard detection, etc. The robot must find the optimal tour between the goal points, minimizing the total travel distance while avoiding obstacles and ensuring that each goal point is visited exactly once.

The warehouse environment constitutes a bounded area representing the warehouse layout with aisles, containing static and dynamic obstacles (e.g., shelves, walls, other robots, or workers). The predefined task points represent locations where the robot must perform specific tasks. The robot is equipped with a camera and ultrasonic sensors for navigation and obstacle avoidance.

The task allocation problem for a mobile robot refers to the process in which the mobile robot departs from the base, visits all task targets (goal points), and then returns to the base with the minimum cost.

The main objectives of the problem discussed are as follows:

- The robot must visit all task points exactly once and complete the associated task at each point;
- The robot must avoid obstacles and follow a collision-free path;
- The total distance traveled should be minimized (this is a TSP with task execution).

This problem involves a combination of path optimization, real-time navigation, and task management.

## 4. Path Planning Framework

### 4.1. Path Planning

Path planning is a critical component in the navigation of autonomous mobile robots. Over the last two decades, significant research efforts [24,25,26,27] have focused on developing path planning strategies for various operational environments. The objective of this process is to determine a collision-free path between two points while optimizing the route in terms of cost. In the case of online path planning, robots rely on sensor data to gather real-time information about their surroundings and build a corresponding map. On the other hand, offline path planning involves the robot obtaining environmental data independently, without the need for real-time sensor input. This process is essential for achieving efficient and reliable autonomous navigation.

To address the motion planning problem for a robot which is requested to operate in a static environment and to visit a set of goal points, we assume that the midpoint G traces a route $R\left(t\right)=\left(x\left(t\right),y\left(t\right)\right)$ represented as a first-degree B-Spline curve:

(1) $$R\left(t\right)=\sum_{i=0}^{K-1}N_{i,1}\left(t\right)p_{i},0\leq t\leq 1$$

where $p_{i}=\{{\tilde{p}}_{0},{\tilde{p}}_{1},\ldots ,{\tilde{p}}_{K-2},{\tilde{p}}_{K-1}\}$ are the control points defined as follows:

- ${\tilde{p}}_{0}={\tilde{p}}_{K-1}$ denotes the depot point;
- $\left\{{\tilde{p}}_{1},\ldots ,{\tilde{p}}_{K-2}\right\}\subseteq (\{Task\;points\}⋃\{intermediate\;points\})$;
- K is the total number of control points;
- $N_{i,1}\left(t\right)$ is the B-Spline basis function;
- The overall number of intermediate points is defined by the user.

The solution of the path planning problem consists of generating the position of the unknown intermediate points such that the overall path is collision-free and has the minimum length L. The length L is calculated by computing the arc length of the image of $R\left(t\right)$ onto the Bump-Surface (Section 3.2). This process incorporates features such as the curve’s curvature (robot’s kinematical characteristics) and robot’s safety (obstacle avoidance). Detailed computations based on specific metrics can be found in [28].

### 4.2. The Bump-Surface Concept

**Figure 2:**
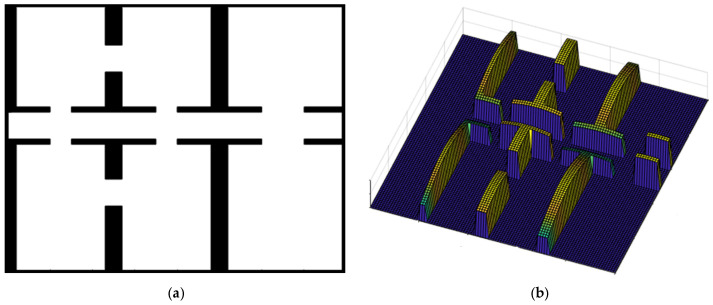
(**a**) The 2D environment layout. (**b**) The Bump-Surface representation.

To address path planning challenges, the Bump-Surface concept [29] is applied as a strategic framework for collision avoidance. This approach is particularly effective for mobile robots navigating 2D static environments. The Bump-Surface technique is based on a network of control points, with an adjustable density to meet specific accuracy requirements for path planning. In essence, increasing the grid density enhances the precision of the planned path. Additionally, the use of B-Spline surfaces provides the flexibility needed for both local and global path control, allowing for the desired level of accuracy. An optimization algorithm is then utilized to search the surface, searching for a collision-free path that satisfies the constraints and goals of the path planning process. Figure 2 presents an illustrative example of the Bump-Surface concept for a mobile robot which is requested to move in a 2D environment cluttered with narrow passages.

## 5. The Developed Approach for Multi-Goal Navigation

### 5.1. The Traveling Salesman Problem (TSP)

The traveling salesman problem (TSP) is a well-known problem in graph theory focused on identifying the most efficient Hamiltonian cycle in which a salesman visits each city exactly once before returning to the starting point [30]. The TSP is one of the most extensively studied problems in combinatorial optimization. The task requires the salesman to visit a finite set of cities, each only once, and then return to the initial city. The goal of optimization is to determine the shortest possible route that the salesman should follow, given the distances between the cities.

The TSP is a fundamental combinatorial optimization challenge that has significant applications in robotics, particularly in path planning, navigation, and task sequencing. In robotic systems, the TSP is often used to optimize the order in which a robot visits a set of locations, minimizing travel distance or time while adhering to specific constraints. Solving the TSP efficiently in robotic applications can enhance operational efficiency, reduce costs, and extend battery life in mobile robots. Moreover, advanced algorithms like heuristic methods, metaheuristics (e.g., GAs, Simulated Annealing), and machine learning approaches are increasingly being integrated into robotic systems to handle large-scale, dynamic TSP instances in real time, addressing challenges such as changing environments and uncertain obstacles.

These developments are critical for autonomous robotics, where quick, adaptive, and optimal solutions are essential for reliable performance. The TSP’s complexity in robotic applications often requires the use of advanced optimization techniques. In the following section, we explore the use of GAs as an effective solution for addressing the TSP in the context of multi-goal robot navigation.

### 5.2. The GA Approach for the TSP

GAs [31,32] are widely recognized metaheuristic methods that have been effectively employed to solve numerous real-world combinatorial optimization problems, demonstrating resilience against local optima. In this study, a GA is designed to determine the optimal sequence of goal points by incorporating the Bump-Surface concept for path planning. The key components are outlined as follows.

Chromosome representation and initialization: A hybrid scheme utilizing both integer and floating-point representations is employed. Each chromosome is divided into two segments: the first segment consists of integers with a length equal to the number of goal points, while the second segment contains floating numbers representing the robot’s unique path on the B-Spline surface. Integer encoding specifically represents the sequence in which the goal points are visited, with each integer corresponding to a distinct goal point. Therefore, the integer segment defines the order in which the mobile robot traverses the goal points. The floating-point segment of the chromosome consists of 2·b real-valued numbers, where b refers to the total number of intermediate points that define the B-Spline curve, and 2 is the dimension of the working space, representing the robot’s path.

**Figure 3:**
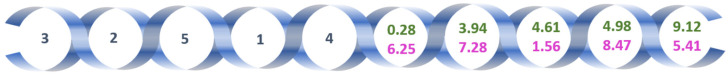
Chromosome hybrid encoding.

For example, consider a scenario where a robot has to serve five goal points starting from its base. Suppose there is one intermediate point between each pair of consecutive goal points, as well as between the base and the (initial or final) goal point, which results in ten unknown variables, corresponding to the coordinates of the intermediate points. In Figure 3, the blue numbers represent the goal points corresponding to the first segment of the chromosome, whereas the green and magenta numbers indicate the x and y coordinates, respectively, corresponding to the second segment of the chromosome.

The chromosome denotes a route starting from the robot base, passing through the goal points 3—2—5—1—4 in that order, and returning to the base. The paths between the five goal points are determined by *b* = 5 intermediate points represented by floating numbers. The first intermediate point (0.28, 6.25) links the base with goal point 3, the second intermediate point (3.94, 7.28) connects goal point 3 with goal point 2, and so on, with the final intermediate point (9.12, 5.41) connecting goal point 4 back to the base.

The initial population is randomly generated, covering the search space with solutions that are uniformly distributed. Each chromosome signifies a potential path for the mobile robot, beginning and ending at its base.

The evaluation strategy: The fitness function reflects the likelihood of a chromosome surviving and reproducing in subsequent generations and is closely linked to the objective function. The fitness function directly corresponds to the objective function, driving the optimization process.

(2) fitness=1L

Selection, crossover, and mutation: The reproduction process involves selecting chromosomes for the next generation, with fitter chromosomes having a higher probability of survival. The roulette wheel selection method is employed, where the probability of selection is proportional to a chromosome’s fitness. Crossover creates new offspring by exchanging genetic material between parents based on the crossover rate, while mutation introduces new genetic variations to maintain diversity. In this study, Order Crossover (OX) is used for crossover, and inversion is applied for mutation [33].

## 6. The Designed Fuzzy Control System for Autonomous Navigation

Fuzzy logic, introduced in [34], is a mathematical framework developed to handle reasoning and decision-making under uncertainty, where information is imprecise or lacks sharp distinctions. In contrast to classical logic, which operates on binary values (true or false), fuzzy logic incorporates degrees of truth, representing values on a continuum between 0 and 1. This is enabled by fuzzy set theory, where elements can belong to a set to varying extents, defined by a membership function. Rather than enforcing strict boundaries, fuzzy sets allow for a gradual transition between membership states, making them particularly suited for the modeling of complex systems with imprecise data, where traditional binary classification fails to capture the inherent uncertainty.

Fuzzy logic theory has found extensive applications in mobile robot navigation, particularly in tasks such as obstacle avoidance and path following. Its strength lies in its ability to manage uncertainty in unstructured environments, enabling robots to respond quickly and effectively while avoiding unforeseen obstacles [35].

In this paper, we propose a fuzzy logic-based methodology that incorporates two key controllers: a fuzzy tracking controller for guiding the robot along a predefined path until it reaches each goal point exactly once and an obstacle avoidance controller for ensuring real-time navigation around both static and dynamic obstacles, allowing the robot to adapt its trajectory and avoid collisions while maintaining efficient movement toward its goal points. The fuzzy logic-based autonomous multi-goal navigation system utilizes the input data provided by the sensors in order to calculate the corrective maneuvers of the mobile robot. The Mamdani fuzzy inference system is used for the implementation of the fuzzy logic systems that output the left and right motor velocities for path tracking and obstacle avoidance. In both fuzzy systems, the “min” operator is applied for rule implication, while the “max” operator is used for aggregation. For defuzzification, the centroid method is used to calculate the motor velocities by determining the center of gravity of the fuzzy set, producing a precise output.

### 6.1. Fuzzy Tracking Controller

**Figure 4:**
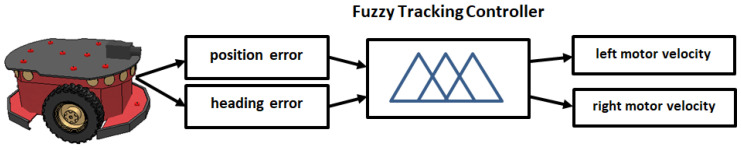
Fuzzy tracking controllers: inputs—position and heading errors; outputs—left and right motor velocities for path tracking.

The primary objective of the robot is to navigate toward a predefined set of inspection points, referred to as goal points, within the environment. If sensor data confirm that no obstacles are present, the robot proceeds directly toward the first goal point (provided by the optimum sequence). A tracking controller is employed to adjust the robot’s heading based on the coordinates of the goal point. The proposed fuzzy tracking controller processes two inputs and generates two outputs (Figure 4). This process is repeated until all goal points are visited by the mobile robot.

**Figure 5:**
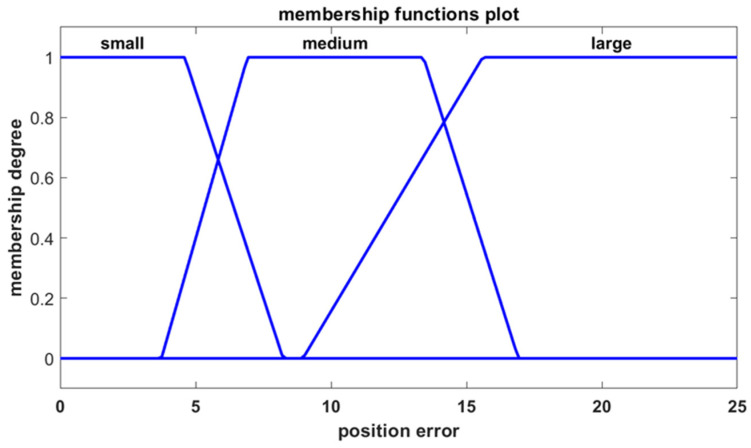
The membership functions for the position error.

**Figure 6:**
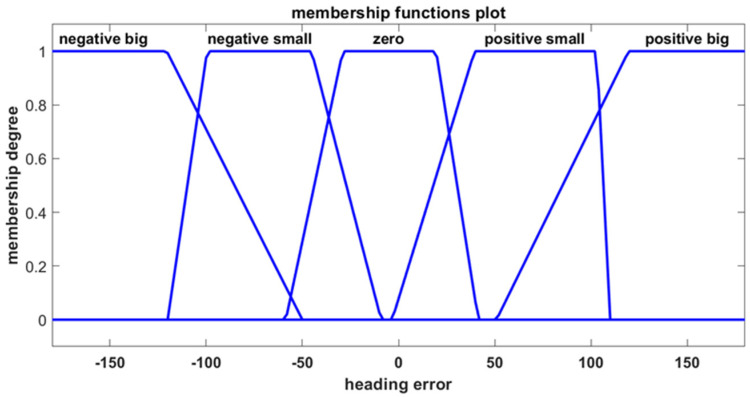
The membership functions for the heading error.

**Figure 7:**
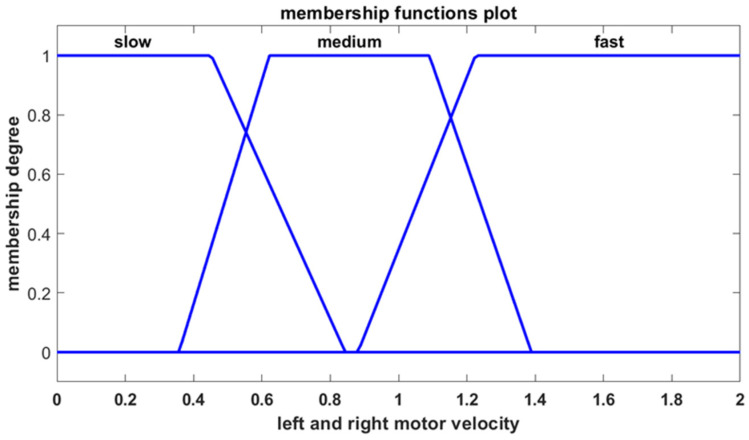
Membership functions for the velocities of the left and right motor.

The first input is the position error, which represents the distance between the robot and the goal point. The second input is the heading error, which reflects the angle between the robot’s velocity vector and the line connecting the robot to the goal point. The position error is described by three trapezoidal membership functions characterized by linguistic terms as shown in Figure 5, while the heading error is defined by five trapezoidal membership functions as presented in Figure 6. The controller produces two outputs, corresponding to the velocities of the left and right motors, with both motor velocities characterized by three trapezoidal membership functions as shown in Figure 7.

**Figure 8:**
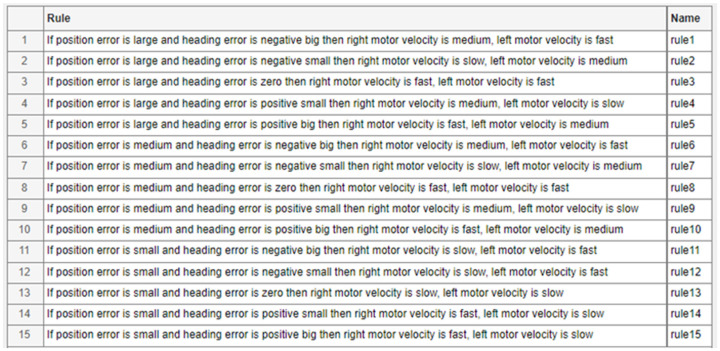
The fuzzy rules for the fuzzy tracking controller.

In total, 15 fuzzy rules are used to describe the relationship between position and heading errors and their impact on the motor velocities. These fuzzy rules are outlined for both the left and right motors in Figure 8. The rules determine that when the goal point lies to the right of the robot’s heading, the robot must turn to the right to adjust its heading. The core principle of multi-goal tracking can be described as follows: if the distance to the target is small and the target is slightly to the right of the robot’s current heading, the robot should make a slow right turn.

**Figure 9:**
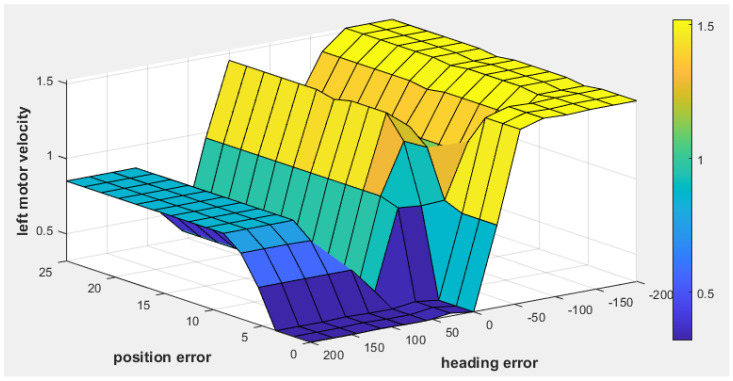
The fuzzy surface plot of the fuzzy tracking controller for left motor velocity.

Figure 9 illustrates the fuzzy surface for the fuzzy tracking controller, showing the relationship between the position error, heading error, and left motor velocity. Warmer colors (yellow) indicate higher activation levels, while cooler colors (blue) represent lower activation levels. As the heading error becomes more negative, indicating the target is to the left, the left motor velocity increases, enabling the robot to turn toward the target. Similarly, higher position error values result in increased motor velocity, ensuring efficient path corrections.

**Figure 10:**
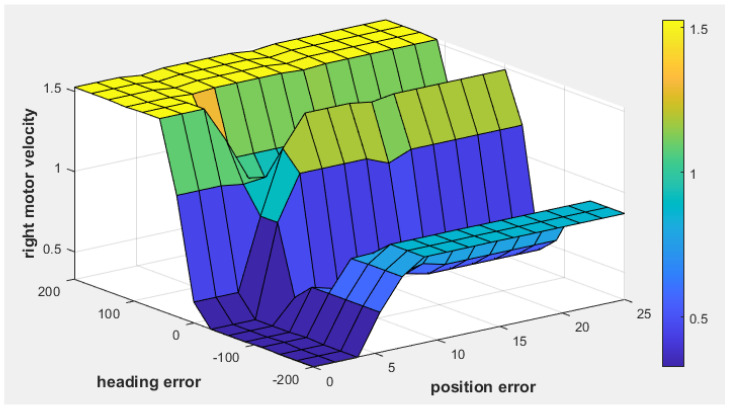
The fuzzy surface plot of the fuzzy tracking controller for right motor velocity.

Similarly, Figure 10 shows the fuzzy surface for right motor velocity. As the position error increases (indicating a greater distance to the target) and the heading error becomes positive (indicating that the target is to the right), the right motor velocity increases to steer the robot toward the target.

### 6.2. Fuzzy Obstacle Avoidance Controller

Obstacle avoidance is a fundamental issue for mobile robots operating in environments with obstacles. This task becomes especially challenging in dynamic environments, where obstacles may exhibit unpredictable movements. An obstacle avoidance controller is triggered when the robot’s sensors detect a nearby obstacle. To mitigate the risk of collision, the robot must steer away in the opposite direction from the detected object. Collision avoidance is assigned the highest priority over all other behaviors, ensuring the robot’s operational safety.

**Figure 11:**
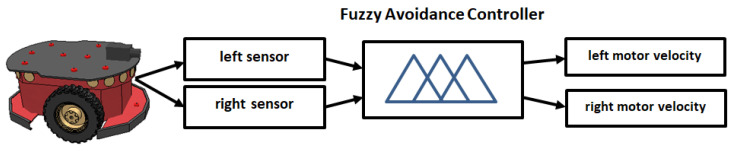
Fuzzy obstacle avoidance controller: inputs—left and right sensors; outputs—left and right motor velocities.

**Figure 12:**
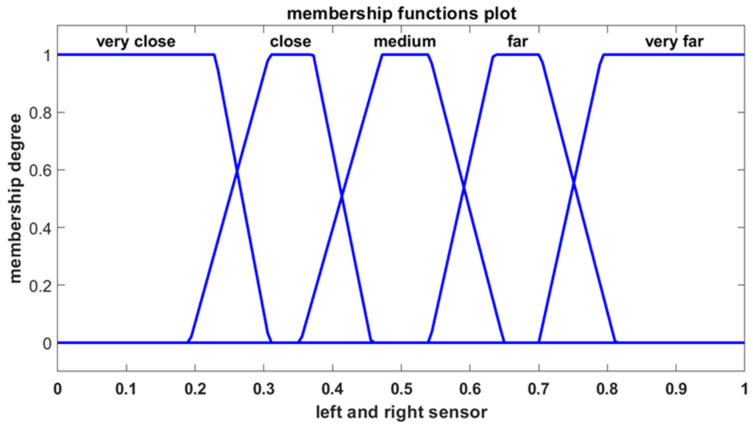
The membership functions for the left and right motor signals.

The fuzzy obstacle avoidance controller operates with two input sensor signals and generates two outputs corresponding to the velocities of the left and right motors (Figure 11). The first input is derived from the left sensor, while the second input is derived from the right sensor. Each of these inputs is represented by five trapezoidal membership functions defined using the linguistic terms shown in Figure 12.

**Figure 13:**
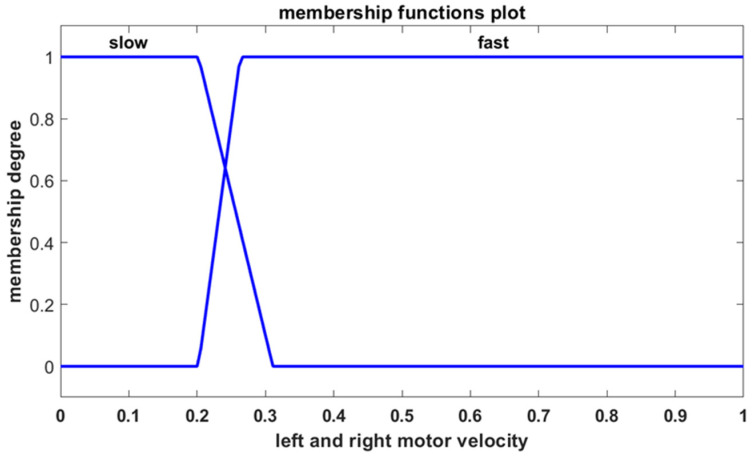
The membership functions for the left and right motor velocities.

The controller’s outputs determine the velocities of the left and right motors. Each motor’s output is characterized by two membership functions, which are defined using linguistic terms, as depicted in Figure 13.

**Figure 14:**
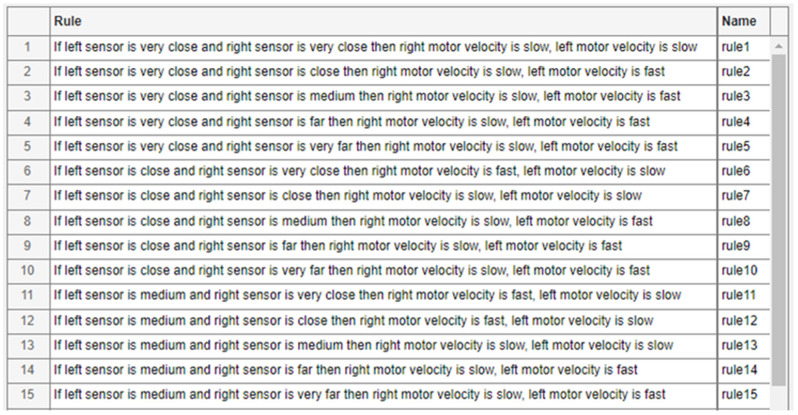
Fifteen out of the twenty-five fuzzy rules for the fuzzy avoidance controller.

A total number of 25 fuzzy rules is established considering all the membership functions of the two inputs (left and right sensor readings). These rules are derived from the observed impact of sensor measurements on motor velocities. The first 15 fuzzy rules for controlling the left and right motors are detailed in Figure 14. The general principle behind adjusting the robot’s orientation is as follows: if the left sensor detects a close object, the robot will steer to the right to avoid the obstacle on the left side.

**Figure 15:**
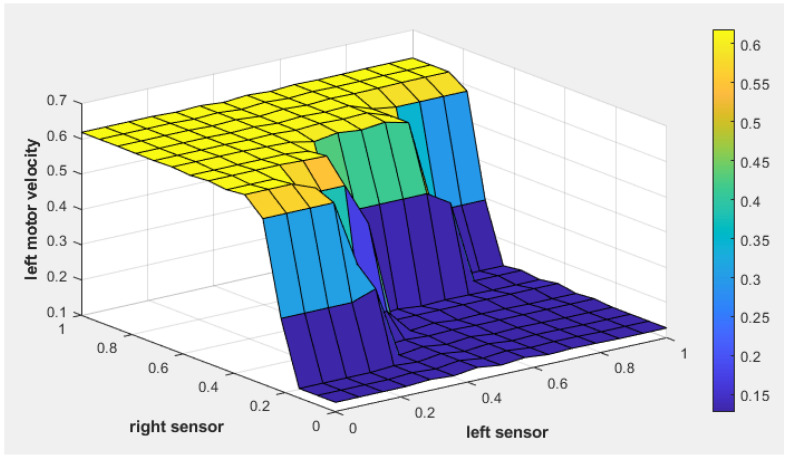
The fuzzy surface of the fuzzy obstacle avoidance controller for left motor velocity.

**Figure 16:**
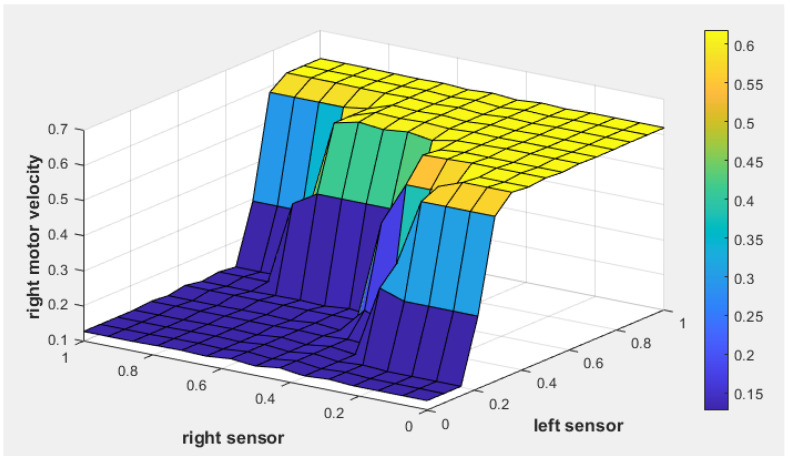
The fuzzy surface of the fuzzy obstacle avoidance controller for right motor velocity.

Figure 15 and Figure 16 illustrate the fuzzy surface plots for the obstacle avoidance controller, showing the relationship between the left and right sensor readings and their impact on motor velocities. The color gradient represents activation levels, with warmer colors, such as yellow, indicating higher activation levels, and cooler colors, like blue, signifying lower activation levels. In Figure 15, as the left sensor detects an obstacle nearby, the left motor’s velocity increases, steering the robot to the right. Similarly, Figure 16 shows that when the right sensor detects a nearby obstacle, the right motor’s velocity increases, steering the robot to the left. These adjustments ensure safe navigation by dynamically altering motor speeds based on sensor inputs.

## 7. Simulation Results for Multi-Goal Autonomous Navigation

The proposed algorithm was programmed with MATLAB and runs in Windows 10 on an Intel(R) Core(TM) i5-7400 CPU @3.00GHz with 8GB of RAM. A detailed analysis of the proposed fuzzy controllers was performed, utilizing the Fuzzy Logic Toolbox in MATLAB R2023a for their design and implementation. To verify the effectiveness of the proposed fuzzy system, a test case is presented in the CoppeliaSim simulation environment. The environment is cluttered with static obstacles and human workers moving in an unpredictable manner (considered moving obstacles).

The mobile robot used in this work is a Pioneer P3-DX, a two-wheel differential drive robot equipped with sixteen ultrasonic sensors—eight at the front and eight at the rear. To simplify the fuzzy obstacle avoidance controller, only the six front sensors were utilized, divided into two sets, enabling the robot to effectively detect and avoid obstacles [36]. For the experiments, it was assumed that the positions of all goal points and static obstacles (racks) are known, but the positions of dynamic obstacles (workers) as well as the additional static obstacles (paper boxes) are unknown to the robot. The main goal was to guide the robot from its base to the specified inspection points exactly once on a smooth path, finding an optimal path through multiple inspection points without colliding with static and moving obstacles, and then return it to its base.

**Figure 17:**
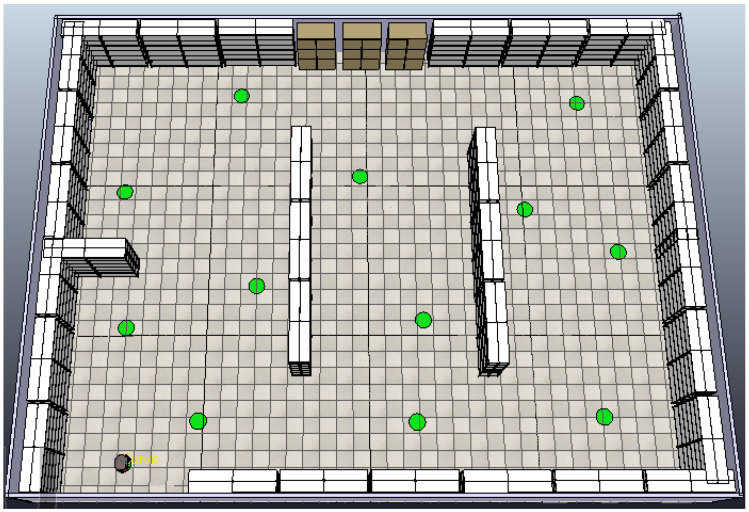
CoppeliaSim scene with static obstacles and inspection goal points (green dots).

Initially, a simulated environment was created in CoppeliaSim, where the base of the mobile robot (depicted as a gray dot) and 13 inspection goal points (green dots) for the robot’s navigation were defined. The initial scene includes only warehouse racks representing the static obstacles, as depicted in Figure 17.

**Figure 18:**
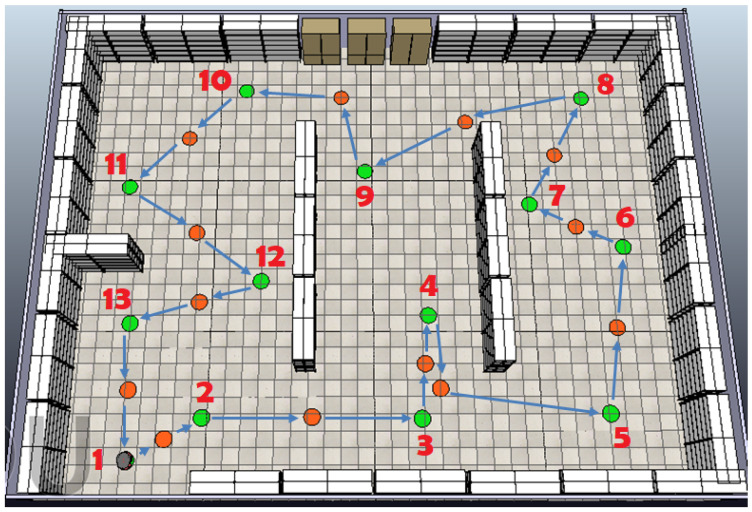
The optimal sequence of goal points yielded by the GA (green: inspection points; orange: intermediate points).

Βased on the static obstacles presented in Figure 17, the optimal sequence is determined using a GA, ensuring the shortest path between the goal points. The collision-free path connecting these points is derived through the Bump-Surface concept, which the robot follows as it moves from one goal point to the next, visiting each point exactly once. To achieve collision avoidance with the static obstacles, a single intermediate point is generated by the GA between consecutive goal points. These intermediate points are strategically determined by the GA to minimize the total path length while ensuring that the trajectory remains collision-free. The resulting optimal sequence 1-2-3-4-5-6-7-8-9-10-11-12-13-1 is depicted in Figure 18, where the intermediate points (represented by orange dots) are placed between consecutive goal points to facilitate the robot in efficiently navigating around warehouse racks and reaching each inspection point. As shown in this figure, the robot starts from its base (numbered as 1), reaches each designated goal point, and then returns to the base.

**Figure 19:**
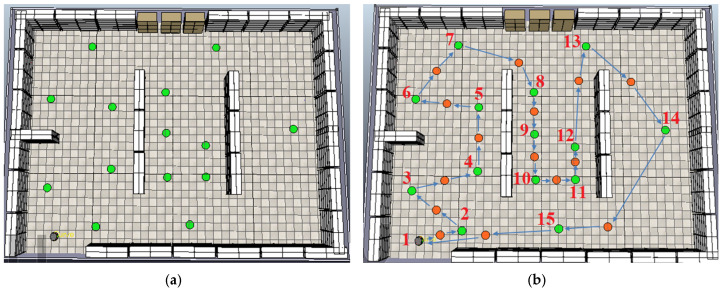
Scene with (**a**) a new arrangement of inspection points (green dots) and (**b**) the reference path generated by the proposed algorithm, optimally connecting the inspection points.

**Figure 20:**
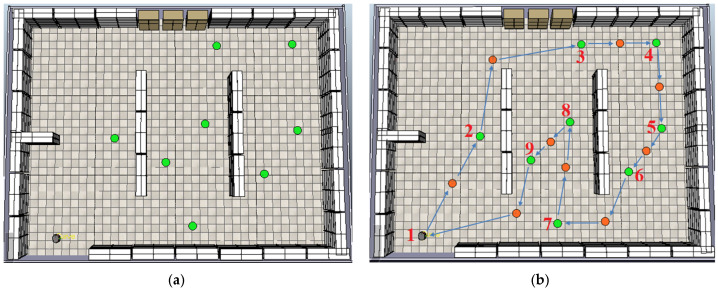
Scene with (**a**) another arrangement of inspection points (green dots). (**b**) Corresponding reference path generated by the proposed algorithm, illustrating the optimal sequence connecting the points.

To further evaluate the proposed path planning strategy, two additional experiments were conducted using different sets of inspection points (green dots), as illustrated in Figure 19 and Figure 20. These experiments included varying numbers of inspection points placed at different locations within the scene, demonstrating the flexibility and adaptability of the proposed methodology in generating reference paths under varying configurations.

These additional experiments highlight the robustness of the proposed path planning strategy in adapting to different configurations of inspection points. By generating reference paths for varying sets of points, the methodology demonstrates its ability to efficiently compute optimal sequences regardless of the spatial arrangement. Next, the analysis shifts to evaluating the robot’s navigation performance in environments with static and dynamic obstacles, starting with the case presented in the first scene depicted in Figure 18.

**Figure 21:**
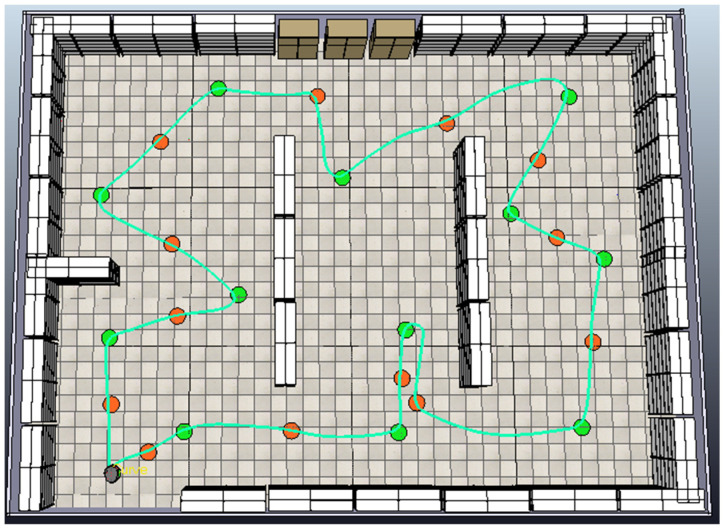
The path the robot follows without the presence of additional obstacles (reference path).

Building on the first example, once the optimal sequence is determined, the robot begins navigating through the environment. Figure 21 illustrates the path the robot follows without the presence of additional obstacles. This path, depicted by the green line, corresponds to the optimal sequence of points generated by the path planning algorithm (reference path). It illustrates the navigation path that the robot takes when guided exclusively by the computed optimal sequence, without additional dynamic or static obstacles. This baseline path serves as a reference for comparing the robot’s performance under obstacle-free conditions and in environments with a variety of static and dynamic obstacles.

**Figure 22:**
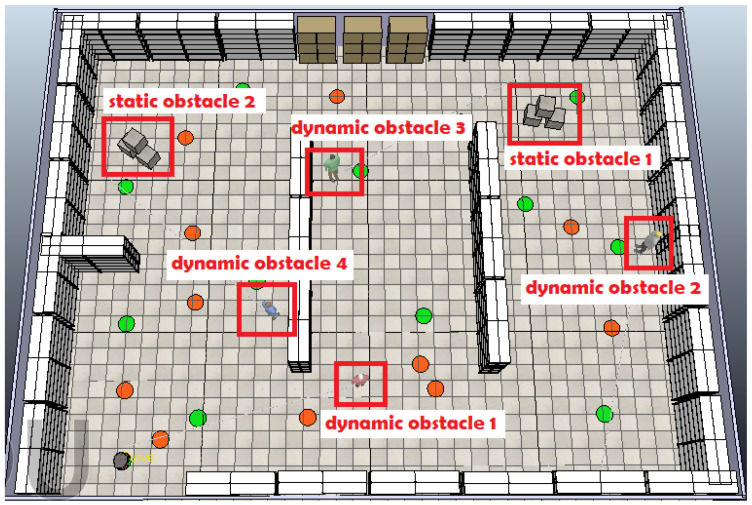
The initial scene including additional static (paper boxes) and dynamic (workers) obstacles.

Moreover, six additional obstacles are introduced into the environment, comprising two static objects (paper boxes) and four moving obstacles (represented by workers walking through the area), as shown in Figure 22. These obstacles present additional challenges to the path planning process, requiring the robot to adjust its trajectory and navigate around them in order to reach the next goal point based on the optimal sequence.

For the robot’s tracking process, a vector P is constructed, containing the (x, y) coordinates of each inspection point that defines the robot’s path from the start (base) to its final destination (base). The robot begins to navigate following a predetermined path through goal points, guided by the fuzzy tracking controller. The controller enables the robot to move from one point in vector P to the next one along the path. When the robot detects an obstacle within a 1 m range, the fuzzy obstacle avoidance controller is activated, causing the robot to temporarily alter its course to avoid the obstacle. Once the robot reaches a goal point, it proceeds toward the subsequent point, repeating this process until it returns to its base.

**Figure 23:**
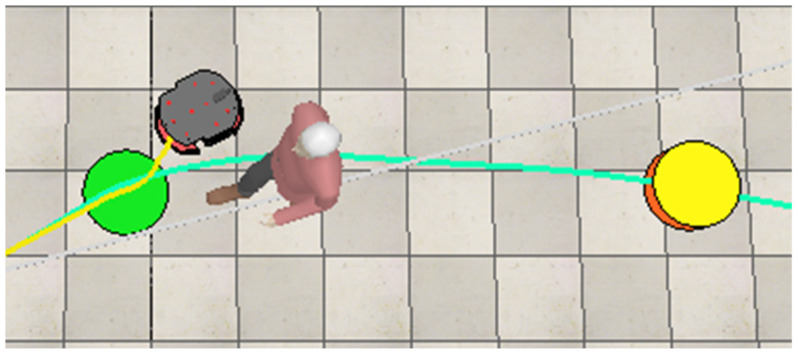
The robot executes a maneuver to avoid a moving worker and reach the next target point (yellow).

In this test case, the first obstacle the robot encounters is a dynamic one, a human worker, which causes the fuzzy obstacle avoidance controllers to activate. The robot adjusts its movement to avoid the human, as shown in Figure 23. In all the figures, the yellow line shows the path the robot takes to move around obstacles and reach the next goal point. Additionally, the yellow dot indicates the next goal point that the robot has to reach.

**Figure 24:**
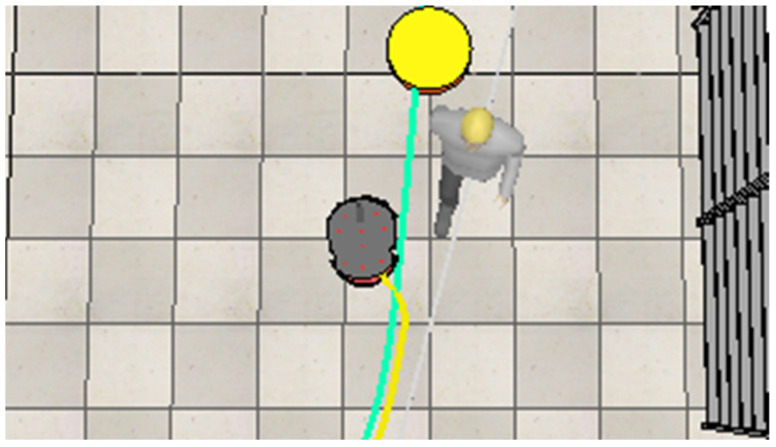
The robot adjusts its path to avoid the second worker and proceed toward the next target point.

After the robot avoids the first worker, it continues on its path toward the next goal point. However, it encounters a second dynamic obstacle, another worker, blocking its path. The robot then performs another maneuver to move around this obstacle, as shown in Figure 24.

**Figure 25:**
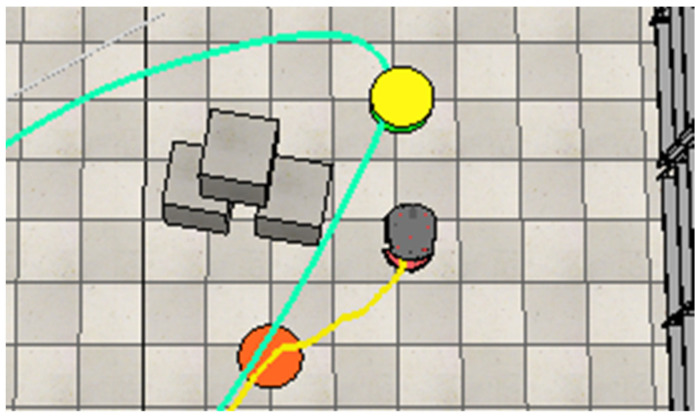
The robot navigates around the paper boxes to maintain a collision-free path.

After avoiding the second human, the robot continues along its path. However, it then encounters its first static obstacle, represented by paper boxes, blocking its way. The robot performs a third maneuver to move around this obstacle, as shown in Figure 25.

**Figure 26:**
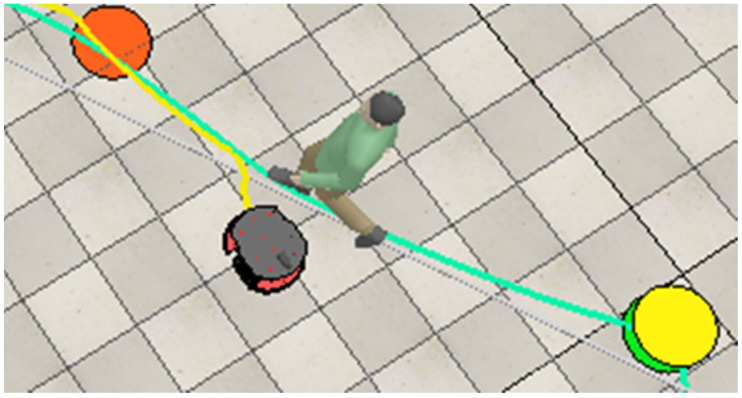
The robot alters its path to avoid the third worker and continue its navigation.

**Figure 27:**
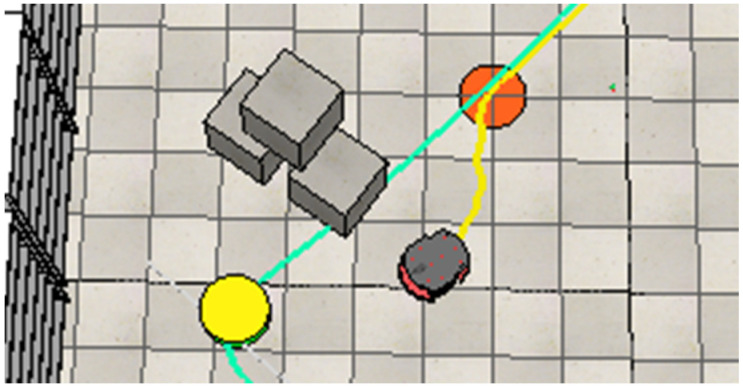
The robot executes a maneuver to avoid the second set of paper boxes.

**Figure 28:**
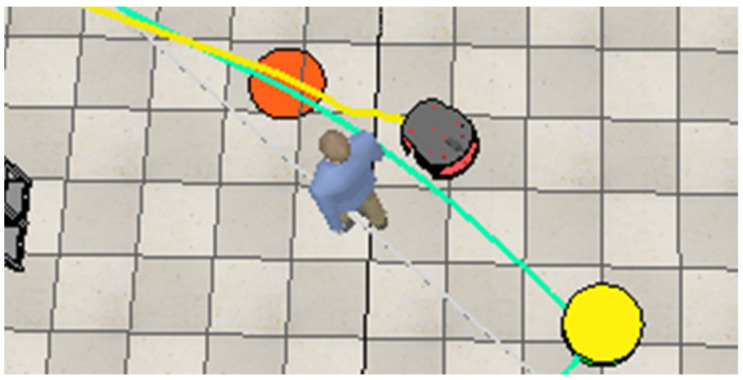
The robot adjusts its trajectory to avoid the fourth worker.

After bypassing the first static obstacle (paper boxes), the robot encounters and avoids the third dynamic obstacle, performing a maneuver to avoid it, as shown in Figure 26. Following this, it navigates around the second static obstacle consisting of paper boxes (Figure 27) and finally avoids the fourth dynamic obstacle (another moving human) (Figure 28).

**Figure 29:**
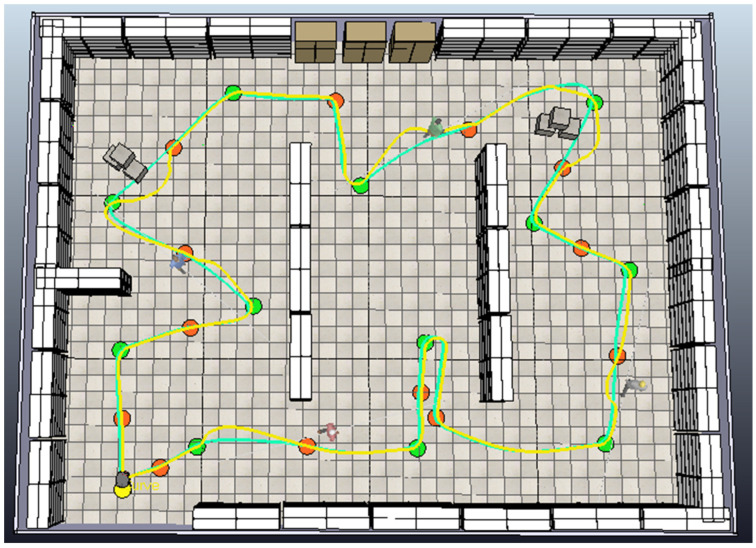
The complete path (yellow) followed by the robot to reach all goal points.

In this process, the robot navigates along a predefined shortest path using a fuzzy tracking controller while simultaneously avoiding unpredictable (static and dynamic) obstacles through a fuzzy obstacle avoidance controller. Figure 29 provides a comparison between two distinct paths. The green line represents the reference path the robot follows in an obstacle-free environment, adhering to the optimal sequence of points generated by the path planning algorithm. The yellow line depicts the robot’s path when navigating through an environment with additional obstacles. This demonstrates the robot’s ability to dynamically adjust its path to avoid both static and dynamic obstacles. By illustrating these two paths together, the figure effectively highlights the robot’s obstacle avoidance capabilities and underscores the differences between the pre-planned reference path and the actual tracked path under real-world conditions. This comparison validates the effectiveness of the proposed fuzzy controllers in ensuring collision-free navigation while achieving the robot’s goals.

**Figure 30:**
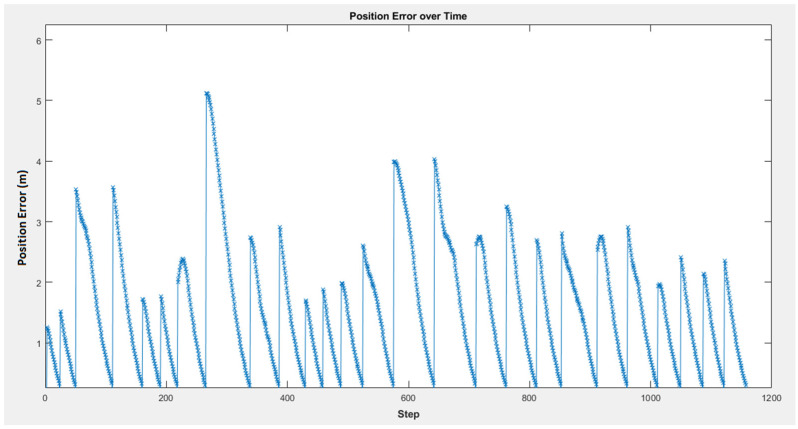
The position error against the number of steps.

Figure 30 illustrates the robot’s position error during its movement between goal points. The position error starts at zero and rises as the robot moves away from its starting point, encountering obstacles or navigating around them. It then decreases back to zero as the robot approaches each goal point. The multiple peaks and valleys in the graph reflect the algorithm’s adjustments, ensuring the robot reaches the goal points with minimal error while avoiding collisions with obstacles. Each drop to zero signifies that the robot has successfully reached one of the goal points. In this figure, there are 26 peaks, corresponding to the 26 goal points that the robot has to reach.

**Figure 31:**
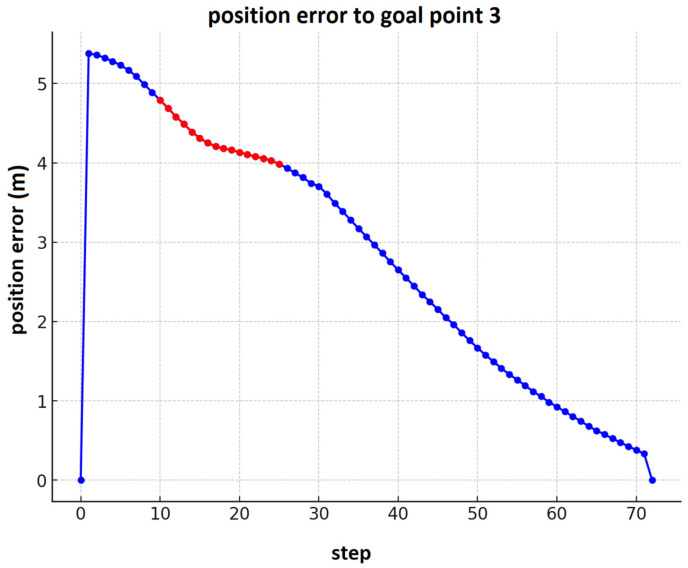
The position error for the robot’s navigation toward goal point 3 with dynamic obstacle avoidance.

Figure 31 illustrates the robot’s position error as it moves toward goal point 3 (presented in Figure 18). The red dots correspond to the time steps when the robot encounters a dynamic obstacle (a worker) in its path. At this stage, the fuzzy obstacle avoidance controller is activated, allowing the robot to make the necessary maneuvers to avoid the obstacle. Once the obstacle is bypassed, the fuzzy tracking controller is re-enabled to guide the robot back on course, reducing the position error as it approaches the second goal point.

**Figure 32:**
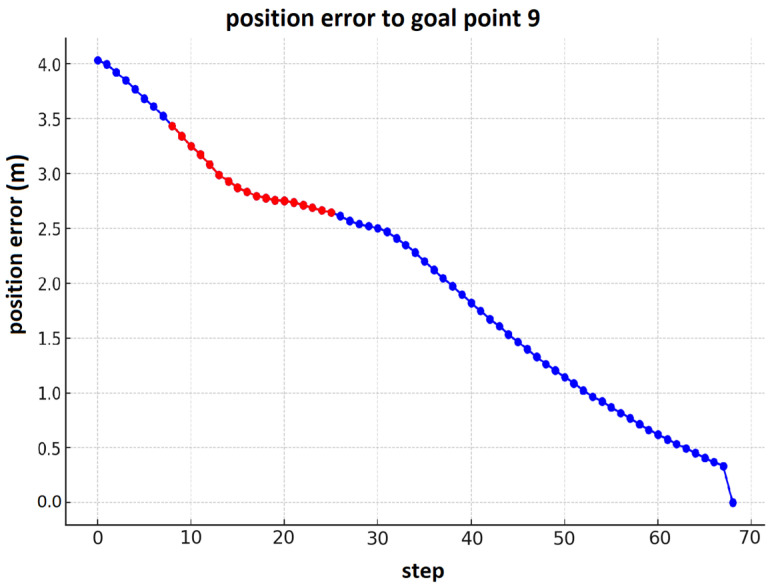
The position error for the robot’s navigation toward goal point 9 with dynamic obstacle avoidance.

Figure 32 illustrates another graph of the robot’s navigation toward goal point 9 (presented in Figure 18). The highlighted section in red shows where the robot encounters its third dynamic obstacle and performs a maneuver to avoid it, before continuing toward the target.

**Figure 33:**
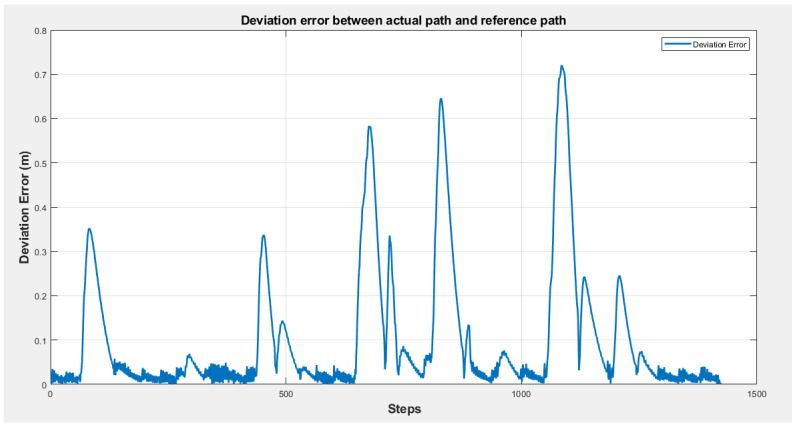
Deviation between the robot’s actual and reference paths.

Figure 33 presents the trajectory error, measured as the deviation between the robot’s actual path and the reference path during navigation. This error quantifies the robot’s performance in maintaining proximity to the planned path while avoiding obstacles. The results indicate that the proposed fuzzy controllers effectively manage path tracking, with a mean deviation of 0.0839 and a standard deviation of 0.1296, reflecting the system’s consistency in real-time adjustments.

The maximum deviation, recorded at 0.7118, corresponds to a critical obstacle interaction where the robot makes significant adjustments to avoid a collision. Despite these deviations, the system quickly reduces the error after each obstacle avoidance event, guiding the robot back toward the reference path. This recovery behavior highlights the robustness of the controllers in handling dynamic obstacles while ensuring efficient navigation.

This test case was evaluated 10 times, with various dynamic objects introduced into the scene, with the robot in each instance starting from a different position and following a distinct path. Notably, the proposed system achieved a 100% success rate across all trials, consistently reaching the endpoint without collisions with either static or dynamic obstacles. This success rate is a crucial indicator of the effectiveness and reliability of the developed navigation algorithms.

## 8. Conclusions

In this work, we presented a novel approach to solving the complex problem of multi-goal robot navigation, which integrates the NP-hard traveling salesman problem (TSP) with a robust path planning algorithm for static obstacle avoidance and two fuzzy controllers for dynamic obstacle handling. Our approach effectively addresses the challenge of determining the shortest collision-free path among multiple goal points while navigating through unpredictable environments with both static and dynamic obstacles. Our method combined a Genetic Algorithm (GA) to optimize the sequence of goals with the Bump-Surface method for path planning so that the robot was able to calculate efficient routes in cluttered environments.

A significant contribution of this study lies in the integration of two fuzzy logic controllers: one dedicated to real-time path tracking and the other to dynamic obstacle avoidance. The fuzzy control system enabled the robot to dynamically adjust its trajectory in response to the unpredictable movement of obstacles, ensuring collision-free navigation. This hybrid strategy combines TSP-based optimization for static environments and fuzzy logic for real-time dynamic obstacle avoidance. Consequently, the developed control strategy not only provides smooth collision-free navigation paths but also adapts to dynamic changes in real time.

The effectiveness of the developed fuzzy control strategy is validated through experimental analysis conducted in the CoppeliaSim simulation environment comprising static and dynamic obstacles. The system’s adaptability to dynamic environments was validated under diverse conditions. In all tested scenarios, the robot successfully avoided collisions and reached all designated goal points, confirming the reliability and adaptability of the proposed system. The experimental analysis shows that the robot consistently achieved efficient, collision-free navigation across various simulated scenarios, demonstrating the robustness of the proposed approach.

Future work will focus on extending this approach to more complex, real-world scenarios involving larger, more dynamic environments and incorporating additional environmental factors such as varying terrain and multi-robot coordination. Additionally, further improvements to the fuzzy controllers could enhance adaptability to highly unpredictable obstacle behavior.

## Data Availability

Data sharing is not applicable.
